# The CRADLE vital signs alert: qualitative evaluation of a novel device designed for use in pregnancy by healthcare workers in low-resource settings

**DOI:** 10.1186/s12978-017-0450-y

**Published:** 2018-01-05

**Authors:** Hannah L. Nathan, Helena Boene, Khatia Munguambe, Esperança Sevene, David Akeju, Olalekan O. Adetoro, Umesh Charanthimath, Mrutyunjaya B. Bellad, Annemarie de Greeff, John Anthony, David R. Hall, Wilhelm Steyn, Marianne Vidler, Peter von Dadelszen, Lucy C. Chappell, Jane Sandall, Andrew H. Shennan, the CLIP Working Group

**Affiliations:** 1 Department of Women and Children’s Health, King’s College London, London, UK; 2 Centro de Investigação em Saúde da Manhiça (CISM), Vila da Manhiça, Moçambique; 3 Universidade Eduardo Mondlane, Faculdade de Medicina, Av. Salvador Allende, 702 R/C, Maputo, Moçambique; 4 Department of Sociology, University of Lagos, Lagos, Nigeria; 5 Centre for Reproductive Health, Sagamu, Ogun State Nigeria; 6 KLE University’s Jawaharlal Nehru Medical College, Belgaum, Karnataka India; 7 Maternity Centre, Groote Schuur Hospital, University of Cape Town, Cape Town, South Africa; 8 Department of Obstetrics and Gynaecology, Tygerberg Hospital, Stellenbosch University, Cape Town, South Africa; 9 Department of Obstetrics and Gynaecology, and the Child and Family Research Institute, University of British Columbia, Vancouver, Canada

**Keywords:** Pregnancy, Blood pressure, Vital signs, Early warning system, Accuracy, Pre-eclampsia, Haemorrhage, Sepsis, Qualitative methods, Low- and middle-income countries

## Abstract

**Background:**

Vital signs measurement can identify pregnant and postpartum women who require urgent treatment or referral. In low-resource settings, healthcare workers have limited access to accurate vital signs measuring devices suitable for their environment and training. The CRADLE Vital Signs Alert (VSA) is a novel device measuring blood pressure and pulse that is accurate in pregnancy and designed for low-resource settings. Its traffic light early warning system alerts healthcare workers to the need for escalation of care for women with hypertension, haemorrhage or sepsis. This study evaluated the usability and acceptability of the CRADLE VSA device.

**Methods:**

Evaluation was conducted in community and primary care settings in India, Mozambique and Nigeria and tertiary hospitals in South Africa. Purposeful sampling was used to convene 155 interviews and six focus groups with healthcare workers using the device (*n* = 205) and pregnant women and their family members (*n* = 41). Interviews and focus groups were conducted in the local language and audio-recorded, transcribed and translated into English for analysis. Thematic analysis was undertaken using an a priori thematic framework, as well as an inductive approach.

**Results:**

Most healthcare workers perceived the CRADLE device to be easy to use and accurate. The traffic lights early warning system was unanimously reported positively, giving healthcare workers confidence with decision-making and a sense of professionalism. However, a minority in South Africa described manual inflation as tiring, particularly when measuring vital signs in obese and hypertensive women (n = 4) and a few South African healthcare workers distrusted the device’s accuracy (*n* = 7). Unanimously, pregnant women liked the CRADLE device. The traffic light early warning system gave women and their families a better understanding of the importance of vital signs in pregnancy and during the postpartum period.

**Conclusion:**

The CRADLE device was well accepted by healthcare workers from a range of countries and levels of facility, including those with no previous vital signs measurement experience. The device motivated women to attend primary care and encouraged them to accept treatment and referral.

## Plain English summary

High blood pressure, bleeding and infection affect women who are pregnant, in labour or have recently given birth. Abnormal blood pressure and pulse measurements can indicate that a woman has one of these conditions and may require treatment. Healthcare workers in low-income countries often lack the equipment and training to measure and understand blood pressure and pulse. The CRADLE Vital Signs Alert is a novel device that accurately measures blood pressure and pulse and is suitable for low-resource settings. Traffic lights within the device help healthcare workers identify women who need additional treatment for these conditions.

This study evaluated whether the CRADLE device was easy-to-use and preferred to other devices. One hundred and fifty five interviews and 6 focus groups involving community- and facility-based healthcare workers and pregnant women and their family members in India, Mozambique, Nigeria and South Africa were conducted. The healthcare workers reported the CRADLE device was easy-to-use. The traffic lights enabled them to make decisions about treatments and made them feel more professional. A minority of healthcare workers in South Africa said the device was tiring to use and questioned its accuracy. The women and their families reported that the traffic lights enabled a better understanding of their health. It encouraged them to attend their appointments and accept treatment. Overall, the CRADLE device was well accepted by healthcare workers and pregnant women from a range of countries and levels of facility.

## Background

Vital signs measurement is one of the most important screening tools in antenatal care, identifying those who require urgent treatment, delivery or referral. Despite its clinical importance, in low-resource settings healthcare workers (HCWs) often have limited access to functioning, accurate devices appropriate for their environment [1]. Even when appropriate equipment is available, the level of training required to accurately measure and act on vital sign measurements (including blood pressure (BP) and pulse) is often lacking. These barriers contribute to avoidable maternal and perinatal mortality and morbidity, which are most prevalent in low-resource settings [2].

The Microlife® CRADLE (Community blood pressure monitoring in Rural Africa & Asia: Detection of underLying pre-Eclampsia and shock) Vital Signs Alert (VSA) is a hand-held, upper-arm, semi-automated device measuring BP and pulse. The device has been accuracy validated in pregnancy, including pre-eclampsia and hypotensive pregnancies, according to formal validation protocols [3, 4]. It meets the World Health Organization’s criteria for low-resource settings, being robust and portable, having low power requirements (charging through a micro-USB port), requiring minimal calibration, and being affordable at $25 USD per unit [5]. The CRADLE VSA has been further modified, incorporating a traffic light early warning system (EWS) [6, 7], aiming to alert HCWs and untrained users to vital sign abnormalities secondary to pre-eclampsia, obstetric haemorrhage and sepsis (the three leading causes of maternal mortality worldwide) [2]. The EWS triggers for hypertension are well-established thresholds of BP used in clinical practice. The EWS triggers for obstetric haemorrhage and sepsis were determined through retrospective analysis of vital signs in women with haemorrhage; shock index (pulse/systolic BP) thresholds were found to be consistently reliable markers of compromise [6, 7]. The predictive ability of the hypertension and shock index EWS thresholds were prospectively validated in women from low- and middle-income countries with either pre-eclampsia, obstetric haemorrhage or sepsis, the results of which are described elsewhere (In Press). Although the current model of the CRADLE VSA does not have mHealth capabilities, incorporation of mHealth technology to future models of the CRADLE VSA is possible (i.e. transmission of vital signs data from the device to mobile phones or directly to a central facility).

By enabling HCWs in low-resource settings to have better access to accurate vital sign measurement, the CRADLE VSA addresses the three delays model of maternal mortality and morbidity [8]. The model is a conceptual framework describing the three delays in receiving care that can result in avoidable maternal mortality and morbidity: (i) delays in the recognition of the maternal health problem and decision to seek care; (ii) delays in reaching the health facility; (iii) delays in the provision of adequate care. The CRADLE VSA aims to identify those who require urgent referral (the second delay) and once at facility, it aims to identify those who require urgent intervention and monitor response to intervention (third delay). In recent years, with improved access to mobile phones, even in the most remote areas of the world, mobile health (mHealth) technologies and other patient monitoring devices are becoming important tools in maternal and perinatal health [9]. Despite improved access to these technologies, formal evaluation of how devices are integrated into routine care [10], contextual evidence on health care workers’ barriers and facilitators and their impact on clinical outcomes is limited [11, 12]. Lessons learnt from a recent large-scale study to improve healthcare in rural India demonstrated the importance of careful evaluation of how innovations might evolve during implementation and how users adapt to the innovations during scale-up [13].

Prior to implementation at scale, it is important to consider the theory of diffusion of innovation, first described by Everett Rogers in 1962 [14], and the five categories of influence, that may be relevant to the implementation of the CRADLE VSA:

- **Relative advantage**, which is the degree to which an innovation is perceived as better than the idea it supersedes (includes economic advantage, convenience and satisfaction)
- **Compatibility** with existing values and practices, which is the degree to which the innovation is perceived as being consistent with the values, past experiences, and needs of potential adopters
- **Simplicity and ease-of-use**
- **Trialability**, which is the degree to which the innovation can be experimented with (a trialable innovation represents less risk to the individual who is considering it)
- **Observable results**: the easier it is for an individual to see the results of an innovation, the more likely they are to be adopted

This qualitative study aimed to determine the usability, feasibility and acceptability of the CRADLE VSA among a variety of users and in diverse socio-economic settings, considering these five clusters of influence. This will inform future device modifications and successful dissemination of the CRADLE VSA for routine use.

## Methods

### Study area

The evaluation was conducted in two separate studies, in the community and primary health centres in multiple study areas in India [15], Mozambique [16] and Nigeria [17], and tertiary hospitals in South Africa. The CRADLE device was developed for use by all levels of HCW. A variety of settings were included in this study to ensure transferability of the findings and relevance for scale-up. Evaluation in India and Mozambique was embedded in the Community Level Intervention for Pre-eclampsia (CLIP) Trial (NCT01911494) [18], a cluster randomised control trial evaluating a package of interventions including the CRADLE device, with evaluation in Nigeria being part of a process evaluation run alongside the trial. Details of each CLIP trial area are described elsewhere [15, 16, 19]. Evaluation in South Africa was embedded in the CRADLE 2 study, a mixed-methods observational study evaluating the CRADLE VSA and the predictive ability of its traffic light EWS. This study took place in tertiary centres, where the rate of adverse outcomes including eclampsia and hysterectomy were high, to effectively evaluate the traffic light thresholds in a high-risk population. These centres cared for women requiring either secondary- or tertiary-level care. In India and Mozambique, a prototype of the CRADLE VSA, with the same accuracy and features suitable for low-resource settings but without the traffic light EWS, was tested; in Nigeria and South Africa, the modified CRADLE VSA, with traffic light EWS, was assessed. For each study, HCWs were trained to use the device through short demonstration and practical sessions. The CLIP trial took place over 33 months (between February 2014 and February 2017). The CRADLE 2 study took place over 15 months (between January 2015 and April 2016).

### Study design

Qualitative evaluation of the CRADLE VSA (and its predecessor: Microlife 3AS1–2) followed the Framework Method [20] to determine the usability, feasibility and acceptability of the CRADLE device and the COnsolidated criteria for REporting Qualitative research (COREQ) Checklist was used to report the methodology and findings [21].

The qualitative evaluation comprised of two phases, the first at 3 months into the studies and the second at 9 months for the CRADLE 2 study in South Africa and 12 months into the CLIP Trial (Table 1). Including two time points allowed for exploration of evolving experience using the device over time [22]. At least 9 months into the study was deemed adequate time for longer-term issues to become apparent. Purposeful sampling was used to identify participants with a broad range of experiences across sites [23]. Snowball sampling was used to ensure views from a variety of pregnant women and family members [24]. CLIP and CRADLE study participants were approached face-to-face and given information sheets specific to the qualitative evaluation and signed an additional consent form to participate in the evaluation.

**Table 1:** Interviews and focus group discussions distribution across sites

Sites:	Mozambique (24 month CLIP trial)	India (33 month CLIP trial)	Nigeria (33 month CLIP trial)	South Africa (12 month CRADLE study)
Device	Prototype CRADLE VSA	Prototype CRADLE VSA	CRADLE VSA	CRADLE VSA
Facility level	Primary health centre and home	Primary health centre and home	Primary health centre	Tertiary hospital
Number of study areas	2	2	2	3
Participants	Agente Communitarios de Saude (APEs)	Auxiliary Nurse Midwives (ANMs) and Accredited Social Health Activists (ASHAs)	Community Health Extension Workers (CHEWs)	Healthcare assistants/students
	Nurses	Nurses	Nurses	Nurses
			Doctors	
	Pregnant women		Pregnant women and family members	
Phase 1				
Timing from start of trial/study	3 months	3 months	3 months	3 months
Number of interviews	29	10	12	25
Number of focus groups	0	0	2	0
Phase 2				
Time from start of trial/study	12 months	12 months	12 months	9 months
Number of interviews	42	10	12	15
Number of focus groups	0	0	2	2

The study comprised of semi-structured interviews [25] and focus group discussions [26] with community- and facility-level HCWs equipped with and trained to use the device and pregnant women and their family members who had come into contact with the device. Community HCWs included Auxiliary Nurse Midwives (ANMs) and Accredited Social Health Activists (ASHAs) in India, Agente Communitarios de Saude (APEs) in Mozambique and Community Health Extension Workers (CHEWs) in Nigeria. At each site, experienced research staff were locally recruited and trained to conduct the interviews and focus group discussions, with guidance from senior social scientists (JS, KM, HB, DA, UC). The research staff were selected due to their familiarity with the community, the CRADLE device and the research question. Each interview and focus group discussion was conducted in the participant’s workplace or home and in either their respective country official language or the local language, according to the participants preference, by one interviewer and was audio-recorded. Participants were approached to participate in either a semi-structured interview or a focus group discussion, with no one participating in both.

### Data management and analysis

All data collection was led by the local team at each site and coordinated by the CRADLE study coordinator and principal investigator in the UK. The field staff transcribed and translated the data to English verbatim. Accuracy was ensured by comparing the audio recording to the transcripts. The data was coded and thematically analysed using NVivo version 10 [QRS, Vic, Australia] by four researchers, both from local sites and coordinating institutions. The four researchers worked independently on four NVivo projects, each with identical coding structures to allow for merging to a single project on completion. The researchers convened regularly (via Skype) to ensure agreement in coding between researchers, discussing emerging themes and to reflect on the findings. The NVivo software coding agreement function (Kappa coefficient) was used to assess agreement between coders. If coding agreement was less than 93%, coding disagreements were discussed during Skype meetings until the researcher group reached a consensus. Factors that may have facilitated or hindered the usability, feasibility and acceptability of the device and those factors likely to influence its potential translation to routine use were explored. While NVivo nodes were created to reflect the emerging themes from participants’ discourse that could be linked to these constructs, the participants and the specific contexts in which such discourses were developed were categorized and labelled by attributes that reflected the age, site, phase of the study, model of device used, years of experience and level of care. Through further coding, similarities and differences within, between and among these groups were compared and common and divergent patterns of responses were explored.

For phase 1, a deductive approach was used, with themes identified through previous researcher experience of device use. For phase 2, additional themes were iteratively determined through researcher consensus in response to emerging themes from phase 1 interviews, using an inductive approach. Field notes were not taken and data saturation was not assessed during the data collection. A large sample size was needed to understand the country context influence in community, primary and tertiary health centres and data saturation was achieved (sample size shown in Table 1).

### Ethical considerations

The study received ethics approval from KLE University, Belgaum India (MDC/IECHSR/2011–12), the CISM Institutional Review Board, Mozambique (CIBS_CISM/08/2013), the Olabisi Onabanjo University Teaching Hospital, Sagamu Nigeria (OOUTH/DA/326/431), University of Stellenbosch, South Africa (N14/06/068), University of Cape town, South Africa (401/2014), University of Free State, South Africa (230408–011), as well as by the UBC C&W Research Ethics Board in Canada (H12–00132).

## Results

In total, 155 interviews and six focus group discussions were conducted in India, Mozambique, Nigeria and South Africa. Interviews included 114 HCW participants and 41 pregnant women and family members (see Tables 1, 2 and 3). Interviews lasted between 30 and 60 min.

**Table 2:** In-depth interview participants’ demographic information

Characteristics	Healthcare worker (%) *n* = 114	Other (%)^a^ *n* = 41
Country		
Mozambique	35 (31%)	36 (88%)
India	20 (18%)	0 (0%)
Nigeria	19 (17%)	5 (12%)
South Africa	40 (35%)	0 (0%)
Facility level		
Community	40 (35%)	NA
Primary health centre	34 (30%)	NA
Hospital	40 (35%)	NA
Age (years)		
15–20	1 (1%)	6 (15%)
21–30	36 (32%)	16 (39%)
31–40	37 (32%)	15 (37%)
41–50	25 (22%)	2 (5%)
51–60	13 (11%)	2 (5%)
Missing	2 (2%)	0 (0)
Gender		
Female	99 (87%)	39 (95%)
Male	14 (12%)	2 (5%)
Missing	1 (1%)	0 (0%)
Years of experience		
0–5	41 (36%)	NA
6–10	38 (33%)	NA
11+	27 (24%)	NA
Missing	8 (1%)	NA

^a^33 pregnant women, 8 family members

**Table 3:** Focus group participants’ demographic information

Characteristics	Healthcare workers (%) *n* = 50
Country	
Nigeria	33 (66%)
South Africa	17 (34%)
Facility level	
Primary health centre	33 (66%)
Hospital	17 (34%)
Age (years)	
15–20	0 (0)
21–30	8 (16%)
31–40	17 (34%)
41–50	18 (36%)
51–60	5 (10%)
60+	2 (4%)
Gender	
Female	50 (100%)
Male	0 (0%)
Years of experience	
0–5	10 (20%)
6–10	10 (20%)
11+	27 (54%)
Missing	3 (6%)

Of the 114 HCWs interviewed, all were either community HCWs, nurses, midwives, or clinical assistants. 35%, 30 and 35% of HCWs interviewed cared for women in their homes, primary health centres and tertiary hospitals, respectively, and 87% of HCWs interviewed were female.

The six focus groups comprised of six to eleven participants. Of the 50 HCWs who took part, all were either community HCWs, nurses, midwives, or clinical assistants; 66% worked in primary health centres and 34% worked in hospital; all HCWs included in the focus groups were female. For more details on participant characteristics see Tables 2 and 3.

Qualitative analysis of the interviews and focus group discussions revealed several major themes across countries and some country-specific themes.

### Healthcare worker opinion on ease-of-use

The view held by most HCWs was that the CRADLE device was easy to use and, for those with prior experience measuring vital signs, preferred to their prior device.

#### Those with previous mercury sphygmomanometry experience

For all HCWs with previous mercury sphygmomanometry experience (the majority), it was agreed that CRADLE device measurement was simpler and quicker. Sphygmomanometry was reportedly technically difficult, often required repeating to achieve a BP reading and the HCW risked appearing inexperienced. Some reported that measurement was now less stressful. Across all sites, HCWs expressed relief not to need a stethoscope for measurement, because it was often difficult to hear the heart sounds in busy environments and it was painful for their ears. In Nigeria, HCWs reported that previously they had not known when to stop inflating the cuff (which, according to the HCW, was painful for the woman), but with the CRADLE device, the sound alerts indicated when to stop inflating. Some favoured the CRADLE device because it gave exact numbers, compared to sphygmomanometry, which required user approximation. One HCW admitted resorting to guessing the BP using sphygmomanometry because it was too challenging, illustrated in the quote below:

*“At times the mercury maybe moving in a scattered manner. So [...] we will not be able to get the accurate reading, we would rely on assumption or guessing by asking the patient if she’s sleeping very well, if she does not have headache, so we may we assume that the patient does not have high blood pressure. That thing affects our work, because at the end of the day the patient may have hypertension.” Focus group discussion, CHEW, Nigeria.*

Unanimously HCWs preferred the simultaneous automated pulse measurement, rather than having to manually measure pulse. For those measuring vital signs in women’s homes, the CRADLE device was smaller, easier to carry and more robust than their prior device. Most community HCWs in India and Mozambique (ASHAs, ANMs, and APEs) had no previous experience measuring vital signs, whereas community HCWs in Nigeria (CHEWS) had previous experience. For those with no previous experience measuring vital signs, it was noted that although they were initially daunted by the new equipment, after simple training, it became easy-to-use.

#### Those with previous automated devices experience

HCWs in the South African hospitals often had previous experience with sophisticated automated devices that could perform repeated measurements automatically and had been used in their departments for many years. The majority, however, reported that they liked the CRADLE device equally or more than the automated devices. For seven HCWs in South Africa, there was a perception of taking a step backwards. Of those, four HCWs described manual inflation as tiring and painful, particularly when measuring vital signs in obese and hypertensive women (requiring more inflation). The same four HCWs also reported BP measurement was slower and that, unlike automated measurement, other tasks could not be done simultaneously. Six of the seven HCWs with negative views, reported confusion about inflation, which would result in errors, particularly in obese and hypertensive women. When probed, the descriptions indicated a lack of understanding of the symbol that represented the need for additional inflation. Of those seven HCWs with predominantly negative views, five were 41–50 years old and five had eleven or more years’ of experience.

### Healthcare worker opinion on the CRADLE device accuracy

In women’s homes and primary health centres in Mozambique, India and Nigeria the CRADLE device was perceived to be accurate. This perception was strengthened over time, demonstrated through a greater proportion of references regarding accuracy in phase 2 compared to phase 1. Accuracy was often assumed due to its “high-tech” and “modern” appearance. One HCW attributed her perceived increase in the incidence of hypertension she was detecting to the device’s accuracy. Another thought the device was accurate because it gave similar readings to a device she used at home.

*“As we are getting more experience we are liking this apparatus more. Now we know that to detect one case of hypertension we have to check everyone* *’* *s BP frequently. As days are passing our faith on machine and accuracy of recording BP has increased tremendously.” Interview, ANM, India.*

In South Africa, views on accuracy varied, with a few South African HCWs distrusting the CRADLE device’s accuracy (*n* = 7). Reasons for doubting the accuracy included variations in BP minute-to-minute and seeing more women with hypertension since using the CRADLE device (a reason for assuming accuracy for others). Seven percent of South African HCWs (2% of all HCWs, *n* = 4), some of whom also questioned BP accuracy, reported that the CRADLE device overestimated pulse in comparison to manual palpation.

The chief nurse at one South African hospital suggested that the accuracy concerns were only in very busy areas of the hospital where staff were dealing with high volumes of patients and struggling with the workload.

*“Now my assumption is that this [CRADLE device] isn't suitable for the high number of patients we have. You know, it's 24/7, a lot of people per day. It seems like the places where people distrust the device the most are the busiest place.” Focus group discussion, Chief nurse, South Africa.*

Another nurse at the same hospital affirmed this view:

*“And it could be a perception, because if you have to pump […] 200 patients, and you don't get accurate readings, […] that makes it hard to believe in these machines, because you don't like it, because you have to work so hard. So, then, the variability is so big. So it might be true, but if you have to pump 3, 4 times for one patient, you get negative from the start.” Focus group discussion, Nurse, South Africa.*

The majority in South Africa trusted the accuracy of the CRADLE device. Some acknowledged that the perceived increase in hypertension may be because their previous devices were underestimating BP. Others acknowledged that occasional unexpected BP readings may have been linked to incorrect technique rather than the device itself.

### Healthcare worker opinion on the CRADLE traffic light EWS

In Nigeria and South Africa, the sites with the CRADLE device with traffic lights, the traffic lights EWS were unanimously perceived positively. Many of those trained to interpret the numbers reported that, although they relied on the numbers, the traffic lights were a useful addition. The traffic lights reassured the HCW on their decision to escalate care, giving them confidence to act quickly. For some, the traffic lights would alert to a problem that would have otherwise been missed. For those with more limited training i.e. community HCWs, the traffic lights were even more useful. These views are illustrated in the quote below:

*“[The traffic lights are] very useful. It’s just like the traffic light we see on the road, because it will tell you when you are supposed to go, when you are supposed to stop, when there is danger sign. So the one seen on [CRADLE device], it will tell okay, this patient is okay, or that you have to refer this patient as fast as possible. So it is very useful.” Interview, Nurse, Nigeria.*

Some reported the traffic lights made counselling of women easier, with the lights being used to illustrate the problem and encourage acceptance of referral or treatment. These views were particularly strong in comparison to sphygmomanometry, a process that restricted involvement of the women in the management of their health.

*“The [traffic lights] have always been of significant help to the midwives. You can easily educate them using the traffic lights on the device and tell them if they need some care. You can even go as far as using another patient with a normal BP as an example.” Interview, Senior nurse, Nigeria.*

### Healthcare worker opinion on impact on work-life

A consistent theme from Mozambique, India and Nigeria HCWs was the sense of professionalism and job satisfaction that the CRADLE device had given them. Many described feeling proud and confident when using the device and become more respected in the community. The device aided communication with senior HCWs and gave them a sense of empowerment to make clinical decisions. These feeling were expressed in the following two quotes:

*“Before we used to get confused about BP so we used to ask our senior doctors to check the BP again as we are getting high BP. Now we are confident in telling our seniors that ‘I have detected one HDP case so please do the needful’.” Interview, ANM, India.*

HCWs reported that the device made it easier to persuade women to be referred when there was a problem. Although the CRADLE device may have encouraged more women to attend primary health centres, they do not associate the device with a heavier workload because the device identified problems earlier, thereby reducing their subsequent workload arising from complications.

*“For me, it has a positive impact […], truly speaking it reduces my workload […]. For example, if someone suffers from high blood pressure and it gives her convulsions. I think it is two times better to work for 20 women rather than only one who gives you a hard time because it is necessary to have medicine to treat the convulsions, we might lose her.” Interview, Nurse, Mozambique.*

In South Africa, although the majority agreed that vital signs measurement was now quicker, 8 HCWs reported that due to perceived errors, difficulties measuring BP in hypertensive women, and inability to do other tasks whilst measuring vital signs, workload had increased.

*“It is actually more time consuming when you need to pump it up because when you are pumping up you could have done something else for your patient, like the temperature reading, or make your patient comfortable or something. And now I have to stand and normally while the blood pressure is reading I speak to the patient to find out how the patient is doing and then we do our notes and we do our recordings.” Interview, Nurse, South Africa.*

### HCW-reported technical issues and errors

In Mozambique and India the CRADLE device was powered by replaceable batteries. Unanimously, HCWs reported that battery life was adequate, lasting 2–3 months depending on intensity of use.

In Nigeria and South Africa, the CRADLE device charged via a micro-USB port. In Nigeria, one charge lasted 2–4 weeks, depending on intensity of use. Charging was dependent on electricity availability, which often resulted in the device being charged when electricity became available, regardless of the battery life. At the South African hospitals, reliable electricity was not a concern. HCWs would charge the device whenever it was not in use and would take it off charge before it was fully charged. For this reason, it was not possible for HCWs to judge how long one charge lasted as it was rarely allowed to run flat. Some reflected this was done out of habit, because it is how they charged their previous automated BP devices and other ward equipment.

On switching on the device, the ‘low battery’ symbol is displayed, as are all other symbols. In Nigeria and South Africa, there was some confusion regarding the ‘low battery’ symbol. When probed, responses suggested confusion between the display shown immediately upon switching on the device and a true ‘low battery’ symbol which flashes throughout vital signs measurement. These findings demonstrate that the display can mislead the user into thinking the device needs charging more frequently than is required and will be fed back to the manufacturers.

Three percent of all HCWs (*n* = 5) reported mechanical issues requiring replacement. Problems included a broken hand pump, broken tubing and three broken charging ports. In South Africa, charging cables (generic micro-USB chargers, also used for most mobile phones) needed to be replaced frequently. Even so, HCWs preferred micro-USB charging to replaceable batteries, as electricity is more readily available than new batteries.

There were very few reports of errors. Some acknowledged that errors were associated with compromised technique e.g. movement. 6% of all HCWs (*n* = 10) thought errors were more frequent when the battery was low rather than considering well-established causes of errors. Many expressed that they appreciated the error alerts on the CRADLE device, explaining that with mercury sphygmomanometry errors occurred without their knowledge. This was illustrated in the quote below:

*“And if we have made any mistake, [the CRADLE device] will show error. If we have made a mistake, we can see the suggestions and repeat it again. In that one you cannot. We don’t realise. We might think that we are not able to hear and so we keep inflating repeatedly.” Interview, ANM, India.*

### Opinions of the women and their families

Unanimously, pregnant women liked the CRADLE device. The device gave them and their families a better understanding of the importance of the measurement of vital signs in pregnant and postpartum women because, unlike sphygmomanometry, they could see the numbers (and the traffic lights, depending on the site). This was reiterated by the HCWs.

*“If they are educated, they see and ask how much [the BP] is. We hold it in front of them and show them. [HCW]: ‘See this is your BP.’ [Patient]: ‘It is good that you come, check and show us. If we go to [the hospital], they don’t tell us anything. They only write it down and give it. Only when we ask them ourselves, they tell us. If we cannot read, we cannot make out. But you explain it to us, come all the way here, check and tell us how much it is.’” Interview, ASHA, India.*

Community HCWs could measure vital signs in the woman’s home using the CRADLE device (previously difficult because mercury sphygmomanometry was less portable). Women and their families saw this as a positive change because they no longer needed to travel to health centres.

Like many HCWs, the pregnant women assumed accuracy due to its digital function and modern look. It was perceived as an improvement in the antenatal service provided at primary health centres and was resulting in more women attending centres. Family members also felt positively about the device, even asking if their vital signs could be measured.

*“My husband liked it, because he felt that the level of care I now receive has improved compared to the ones I received during the previous pregnancies. He was happy and prayed for those who introduced the [CRADLE] device.” Interview Pregnant woman, Nigeria.*

## Discussion

These findings demonstrate that the CRADLE device was easy-to-use by all levels of HCW and well accepted by the vast majority, despite some having never measured vital signs previously. Technical modifications, including the traffic light early warning system and micro-USB charging, to ensure suitability for community HCWs in low-resource settings were of benefit to users. The traffic light EWS was appreciated by all, even those with prior training to respond to vital sign thresholds.

The findings also highlighted minor technical issues that need to be addressed prior to scale-up, either by requesting manufacturing adaptations or by improving device training.

Strengths of this study include the range of countries, levels of facility, HCWs experience and training and the different studies hosting the evaluation. A sufficient number of interviews and focus groups ensured theoretical saturation and having two phases of data collection (at 3 months and 9 or 12 months) allowed for temporal variations to be explored. The study gives an extensive and diverse range of views increasing the likelihood of transferability. In addition, all data was collected by local researchers with familiarity of the region and socio-cultural context, some of whom also participated in the analysis.

A limitation of the study is the potential impact of social desirability bias. Participants were aware of their role in the study and the objective to explore their views related to the CRADLE device. Participants may have perceived the researcher as a person of power, aiming to portray the CRADLE device as a success, which may have influenced participant responses. To minimise this, specific instructions regarding the purpose and confidentiality of the interviews were explained.

The influence of researcher reflexivity was considered [27, 28]. Some of the researchers worked on other aspects of the study and had extensive understanding of the CRADLE device. Throughout the research process, the researchers considered their own opinions of the device, their relationship with the participants and the influence on the data generated. Although they may have taken a different emphasis from that of independent observers, detailed exploration of the facilitators and barriers was possible due to a good understanding of the intricacies of the device.

The majority of studies evaluating BP devices focus on accuracy, describing quantitative validations. Very few studies have evaluated the usability and acceptability of devices, or assessed the problem from the user’s perspective rather than the developer’s perspective, these being critical for successful implementation. The only published studies to qualitatively evaluate BP devices in the hands of users are studies of home BP monitoring and telehealth in high-income countries, exploring the use of BP devices in the hands of patients rather than HCWs [29–32]. This study is unique in that it explores the opinions of a variety of HCWs from a number of low- and middle-income countries.

The study findings can be explained following the diffusion of innovation model and the impact of the five categories of influence the model describes [14].

### Relative advantage, simplicity and ease-of-use

In Mozambique, India and Nigeria, the CRADLE device was perceived as a welcomed resource improvement compatible with existing routines, despite differences in level of experience, training, and working-environment across sites. Their previous BP device experience was more varied, with some having used automated devices but most having only used mercury sphygmomanometry. The CRADLE device was perceived as more technologically advanced and easier-to-use than their previous equipment and accuracy was presumed.

### Observable results

In Mozambique, India and Nigeria, the device improved confidence and job satisfaction for many HCWs. The device empowered lower cadres of HCW to undertake their work with confidence and the traffic lights were reported to improve escalation of care for all cadres of HCW. The traffic lights also gave women a better understanding of their health and the need for vital signs monitoring. This led to the perception that more women chose to attend primary health centres and easier counselling of women who needed urgent treatment or referral.

### Compatibility with existing values and practices

Across the sites, HCWs reported that although more women attended health centres because of the device, this did not negatively impact on their workload as the device enabled complications to be identified and treated promptly. Rather than perceiving the immediate burden of having more women to attend to, these HCWs were valuing the longer-term gains of the device. This is an important finding, which can be utilised when transmitting the value of the CRADLE device to other HCWs at scale-up.

In South Africa, although the majority thought positively, a small proportion of HCWs disliked the CRADLE device and questioned its accuracy. These HCWs worked in very busy units, measuring vital signs many times a day, having previously relied on sophisticated (but necessarily validated or accurate) automated machines. Manual inflation with the CRADLE replacement was perceived as a step backwards and resulted in resentment of the CRADLE device. Although the CRADLE device is more accurate than their existing devices, the HCWs associated their sophisticated machines with better accuracy. Their existing workload and previous device experience influenced their opinions. This lack of awareness of device accuracy can be described as a barrier to successful uptake [33]. Of note, they questioned CRADLE device accuracy specifically in those requiring more manual inflation (obese and hypertensive women). BP measurement can be more challenging in these women. These isolated negative experiences can impact on successful implementation and can be difficult to overcome [33, 34].

In South Africa, older age emerged as a barrier to adoption in this study, perhaps due to resistance to change rather than fear of technological complexity. The younger respondents were more adaptable to the novel technology, whereas the older respondents were comfortable with their existing devices and resistant to change in their routines. Age has also been identified as a barrier to the uptake of technology in other studies [33, 35]. Older users may need additional training and support to improve adoption.

Across the Mozambique, India and Nigeria sites and at two of the three South African sites, there was a strong researcher presence and support system. At these sites, research staff could assist and reassure HCWs using the new technology and educate on the importance of accurate devices. Successful implementation at the other South African site may have been hindered by the limited technical support. Similar barriers have been demonstrated in other studies evaluating the implementation of newly adopted health technologies [33].

### Implications for clinicians and policy-makers

This study has demonstrated the importance of training and support when implementing a new technology. It has also highlighted aspects of the CRADLE device that require more focused education (accuracy, inflation, charging). Considering these findings, a simple training package that is transferable across settings and complements the simplicity of the device is designed for scale-up.

The perception of the user plays a crucial role in the innovation process [34]. This study has shown that existing workload and previous experience should be considered as context-dependent barriers and facilitators to implementation at scale. The study suggests that in relatively busy and well-resourced areas, such as South African tertiary centres, fully-automated vital signs devices, that automatically and regularly repeat readings, may be more appropriate, if accuracy has been confirmed. The traffic light EWS, however, remains a useful component even amongst trained staff. An automated version of the CRADLE device may be better suited in these settings.

The CRADLE VSA was designed for use by community HCWs to identify pregnant and postpartum women who require urgent intervention and referral. When considering the CRADLE device in the context of the three delays model of emergency obstetric care, it was designed to address the second and third delays (identifying and reaching health facility and receiving adequate and appropriate treatment) [8]. Reflecting this, HCWs reported that the device enabled identification of women needing intervention, who might have otherwise been missed and that the traffic light EWS encouraged the decision for escalation of care. This device has the potential to strengthen task-sharing approaches and may improve the quality and safety of care women receive. An unanticipated finding from our study was that the CRADLE VSA could also address the first delay (deciding to seek care). Women were motivated to attend because of the new technology. The visual traffic light triggers gave the women a better understanding of the importance of screening and assisted in explaining the meaning of abnormalities and encouraged acceptance of life-saving treatments and referral to higher-level facilities. A major influencer of poor antenatal care attendance is experience of poor-quality care [30, 36]. The introduction of the CRADLE VSA to facilities may enhance perceptions of high quality care and encourage better antenatal attendance, which would be of interest to policy-makers.

## Conclusions

This study enabled better understanding of the facilitators and barriers to use of the CRADLE VSA. The CRADLE device was well accepted by healthcare workers from a range of countries and levels of facility, including those with no previous vital signs measurement experience. The device motivated women to attend primary care and encouraged them to accept treatment and referral. These findings could be used to guide scale-up.

The device was designed to identify women who are at risk of avoidable maternal morbidity and mortality and prompt escalation of care. Although this study demonstrated that the device is acceptable and of benefit to HCWs, it is not known whether this will be translated to reductions in maternal mortality and morbidity. The CRADLE 3 Trial is underway across ten low-income country sites to determine the impact that the CRADLE VSA and a simple training package implemented at community, clinic and hospital level, has on maternal mortality and severe morbidity.

## Abbreviations

ANMs: Auxiliary Nurse Midwives
APEs: Agente Communitarios de Saude
ASHAs: Accredited Social Health Activists
BP: Blood pressure
CHEWs: Community Health Extension Workers
CLIP: Community Level Intervention for Pre-eclampsia
CRADLE: Community blood pressure monitoring in Rural Africa & Asia: Detection of underLying pre-Eclampsia and shock)
EWS: Early warning system
HCWs: Healthcare workers
VSA: Vital Signs Alert
